# Chemically Modified Soluble Starches as Green Scale Inhibitors in Petroleum Production

**DOI:** 10.1021/acsomega.5c05924

**Published:** 2025-12-31

**Authors:** Erika M. Da Silva, Tatiana S. L. Maravilha, Ronald W. P. Ortiz, Allan Belati, Ana B. O. Souza, Fabricio Venancio, Evelin A. Manoel, Vinicius Ottonio O. Gonçalves, Tiago Cavalcante Freitas, Jussara M. Silva, Monica T. da Silva, Rosane A. Fontes, Vinicius Kartnaller, João Cajaiba

**Affiliations:** † Núcleo de Desenvolvimento de Processos e Análises Químicas em Tempo Real, 28125 Instituto de Química, Universidade Federal do Rio de Janeiro, Polo de Xistoquímica, Rua Hélio de Almeida 40, Cidade Universitária, 21941-614 Rio de Janeiro, Brazil; ‡ Laboratório de Tecnologia de Materiais Poliméricos, Divisão de Materiais, Instituto Nacional de Tecnologia, Avenida Venezuela 82, Saude, 20081-312 Rio de Janeiro, Brazil; § Departamento de Bioquímica, 341411 Instituto de Química, Universidade Federal do Rio de Janeiro, Avenida Athos da Silveira Ramos 149, Cidade Universitaria, 21941-611 Rio de Janeiro, Brazil; ∥ 125096 Centro de Pesquisas da Petrobras Leopoldo Americo Migues de Mello, Avenida Horácio Macedo 950, Cidade Universitaria, 21941-915 Rio de Janeiro, Brazil; ⊥ Laboratório de Análise e Desenvolvimento para Estudos em Energia e Sustentabilidade, Instituto de Química, Universidade Federal do Rio de Janeiro, Polo de Química, Bloco C 1281, Avenida Horácio Macedo, Cidade Universitária, 21941-598 Rio de Janeiro, Brazil

## Abstract

The development of green scale inhibitors is crucial for the petroleum industry. This work investigates chemically modified starches as green alternatives for scale control. Soluble starch was modified through two routes: carboxymethylation with chloroacetic acid and esterification with maleic anhydride. The modifications were confirmed by infrared spectroscopy, and degrees of substitution were quantified by titration. Tube-blocking tests demonstrated that both modified starches markedly improved calcium carbonate inhibition, reducing the minimum inhibitory concentration from 500 mg L^–1^ for unmodified starch to 150 mg L^–1^ for carboxymethyl starch and 125 mg L^–1^ for starch maleate. The superior performance of starch maleate can be attributed to its higher degree of substitution and presumable steric effects. Scanning electron microscopy and X-ray diffraction analysis revealed that carboxymethyl starch promoted distortion of both the shape and size of calcium carbonate solids, while starch maleate more effectively reduced particle size. These morphological modifications were more pronounced than those induced by unmodified starch, contributing to the enhanced inhibitory performance. In addition to superior performance, the maleic anhydride esterification route offers a simpler and more sustainable modification process, as it proceeds under solvent-free conditions and eliminates neutralization and purification steps. Overall, these results demonstrate that chemically modified starches, particularly starch maleate, are promising candidates for effective and sustainable scale inhibition in petroleum facilities.

## Graphical abstract

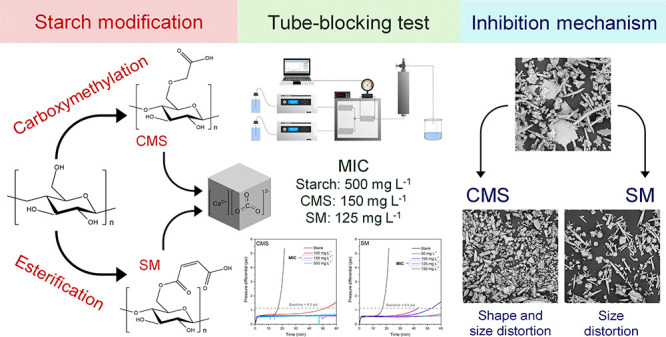

## Introduction

Inorganic scale formation is a major challenge for flow assurance in the oil and gas industry. These deposits can reduce system productivity and, in severe cases, cause production shutdowns by clogging critical components such as production and injection wells, pipelines, downhole equipment, and surface facilities.^1^ Scale forms when the concentration of inorganic salts in produced water exceeds their solubility limits, leading to precipitation and adhesion to surfaces as solid deposits. The most common inorganic scales encountered in petroleum production are barium sulfate, calcium carbonate, and strontium sulfate.^2^ To mitigate scale-related issues, the industry relies on three main strategies: mechanical removal, chemical dissolution, and the application of scale inhibitors.^3^

Scale inhibitors are chemical additives that interfere with one or more of the steps of the crystallization process, including nucleation, crystal growth retardation, and distortion of the size and morphology of the precipitated solids. Although inhibition does not completely prevent scale formation, it significantly delays scale deposition, making it an effective method for flow assurance.^4^ The inhibitors can influence both the thermodynamics and kinetics aspects of scale deposition. While thermodynamics predicts the tendency of a system to form scale, kinetics determines the rates at which crystals nucleate, grow, aggregate, and adhere on surfaces. In other words, thermodynamics defines the potential for scale formation, whereas kinetics defines its operational impact. The kinetics govern not only the rate of precipitation but also the morphology and particle size distribution of the resulting solids. Even when a brine is thermodynamically supersaturated with respect to a given mineral, slow kinetics may significantly delay or prevent deposition during production.^4,5^

Commercially available scale inhibitors include phosphonic acid derivatives and polymers, where phosphonates are generally more effective than polymers. However, a major drawback of phosphonates is their phosphorus content, which, if improperly discharged, can contribute to eutrophication. Consequently, despite their efficacy, environmental concerns have driven research into alternative solutions such as green scale inhibitors.^3,4,6^ In turn, polymers offer the advantage of acting through multiple inhibition mechanisms and at various stages of scale formation, depending on the functional groups present and their interaction with scaling ions.^7–9^ However, commonly used polymeric inhibitors, such as poly­(acrylic acid), poly­(methacrylic acid), poly­(vinylsulfonic acid), poly­(maleic acid) and their copolymers, exhibit poor biodegradability, raising additional environmental concerns.^10,11^

Research into green scale inhibitors has explored a range of materials, including plant extracts,^12,13^ biodegradable organic molecules,^14,15^ polymers synthesized from biodegradable organic molecules,^16^ naturally occurring biodegradable polymers,^17^ and chemically modified biodegradable polymers.^18,19^ To be considered ‘green’, these inhibitors must be biodegradable, nonbioaccumulative, and nontoxic.^20^ Natural polymers (biopolymers) are of particular interest due to their environmental compatibility, abundance, and availability. They can also be chemically modified to enhance their performance by lowering the minimum inhibitory concentration, improving solubility, increasing thermal stability, and extending shelf life. Moreover, natural polymers can be used in the synthesis of copolymers to increase the biodegradability of conventional polymeric scale inhibitors. Table 1 summarizes studies on the application of natural biodegradable polymers and their derivatives for scale inhibition.

**Table 1:** Studies on the Application of Biodegradable Polymers in Scale Inhibition

**polymer**	**scale**	**reference**
natural biodegradable polymers		
chitosan and alginate	CaSO_4_	Khamis et al.^21^
guar and xanthan gums	CaCO_3_, CaSO_4_	ElKholey et al.^17^
inulin	CaCO_3_	Mahmoodi et al.^22^
soluble starch	CaCO_3_	Ortiz et al.^23^
soluble starch	CaCO_3_	Oliveira et al.^24^
modified biodegradable polymers		
carboxymethyl cellulose	CaCO_3_	Yu and Yang^25^
carboxymethyl cellulose and carboxymethyl starch	CaSO_4_	Saleah and Basta^26^
carboxymethyl cellulose and hydroxyethyl cellulose	CaCO_3_	Fernandes et al.^27^
carboxymethyl inulin	CaCO_3_	Zhang et al.^28^
carboxymethyl starch	CaCO_3_	Wang et al.^19^
oxidized lignosulfonate	CaCO_3_	Ganguly et al.^10^
phosphonated chitosan	CaCO_3_, BaSO_4_, SrSO_4_	Mady et al.^29^
copolymers		
chitosan-acrylic acid-polysuccinimide	CaCO_3_	Zheng et al.^30^
chitosan-maleic anhydride-styrene sulfonic sodium-acrylamide	CaCO_3_	Guo et al.^31^
chitosan vanillin Schiff base	CaCO_3_	Ramanathan et al.^32^
carboxilated chitosan-polysuccinimide	municipal wastewater	Gao et al.^33^
carboxymethyl cellulose-*graft*-poly(acrylic acid)	CaCO_3_	Yu and Yang^25^
N,O-carboxymethyl chitosan	CaCO_3_	Baari et al.^34^
pectin-poly(acrylamide)	CaSO_4_	Chauhan et al.^35^
poly(aspartic acid)-oxidized starch	CaCO_3_, CaSO_4_	Chen et al.^36^
starch-*graft*-poly(acrylic acid)	CaCO_3_	Yu et al.^37^

Most of the studies listed in Table 1 focus on water treatment systems. Therefore, it is important to evaluate the potential of these biopolymers and their derivatives under conditions relevant to the petroleum industry (e.g., dynamic flow, high-temperature, high-pressure, high-salinity conditions, complex aqueous and oleos matrix). One such biopolymer is starch, which has been investigated in modified forms such as carboxymethyl starch and as a copolymer with poly­(acrylic acid).^19,37^ Starch is of interest due to its low cost, high availability, and derivation from agricultural residues and agro-industrial byproducts. Indeed, there are previous works that evaluated the potential of soluble starch and starch-rich aqueous extracts for calcium carbonate scale inhibition. In one study, aqueous potato extract was found to influence both the kinetics and equilibrium of calcium carbonate precipitation. The solids formed in the presence of this extract were calcite crystals with distorted rod-like morphology.^23^ In a subsequent study, aqueous sweet potato extract demonstrated effective scale inhibition at a concentration of 500 mg L^–1^ at 80 °C under dynamic tube-blocking test conditions.^24^ Starch-rich extracts obtained from various sources, such as barley, sweet potato, ginger, and rye, have also been investigated. Compatibility tests revealed that the barley extract exhibited the highest compatibility, while rye extract showed incompatibility at higher concentrations.^6^

Native starch offers several advantages, including wide availability, low cost, renewability, and biodegradability. However, it also exhibits functional limitations such as low water solubility, rapid retrogradation, syneresis, low thermal stability, and poor shear resistance. To address these limitations and give properties suitable for specific industrial applications, various modification strategies have been developed. These modifications are generally classified into three main categories: chemical, enzymatic, and physical.^38^ Chemical modification of native starch is particularly attractive in the context of scale inhibition, as it allows the introduction of functional groups necessary for interaction with scaling ions and solids through various inhibition mechanisms. Whang et al. synthesized carboxymethyl starch (CMS) with different degrees of substitution and molecular weights. Their results showed that a higher degree of substitution of carboxymethyl groups and a lower molecular weight favored the distortion of calcium carbonate crystal growth and improved scale inhibition efficiency in static tests.^19^ Yu et al. synthesized starch-*graft*-poly­(acrylic acid) with different grafting ratios and grafted-chain distributions. They found that the copolymer with a relatively low grafting ratio, but a higher number of grafted chains exhibited superior antiscaling performance. This improvement was attributed to a synergistic effect between adjacent poly­(acrylic acid) side chains, which increased the chelation and dispersion activities of this copolymer.^37^

To the best of our knowledge, the studies by Whang et al. and by Yu et al. are the only prior reports on starch modification for scale inhibition. However, these investigations focused on water treatment applications, employing static tests conducted at approximately 70 °C, atmospheric pressure, and low salinity. Consequently, chemically modified starches have not yet been systematically evaluated as scale inhibitors under dynamic, high salinity, high temperature, and high-pressure conditions, characteristic of petroleum production. In this work, we investigate the scale inhibition performance of two chemically modified soluble starches under conditions relevant for petroleum production. Soluble starch was subjected to two types of chemical modifications: carboxymethylation and esterification. Both modified products, CMS and starch maleate (SM), demonstrated promising inhibition performance in tube-blocking tests conducted under conditions relevant to petroleum production. Moreover, the esterification method using maleic anhydride offers practical advantages, as it does not require the use of solvents and additional neutralization and purification steps.

## Materials and Methods

### Materials

Soluble starch with molecular weight of 342.30 g mol^–1^ was purchased from ACS Cientifica (Sumare, Brazil). Chloroacetic acid, sodium hydroxide, hydrochloric acid, ethanol, calcium chloride, sodium bicarbonate, sodium chloride, and maleic anhydride were purchased from Isofar (Rio de Janeiro, Brazil). All reagents were of analytical grade and used as received. Carbon dioxide was supplied by Air Products (Rio de Janeiro, Brazil). Ultrapure deionized water Type I water was used for the preparation of aqueous solutions.

### Starch Modifications

Figure 1 presents the two modification routes of soluble starch: carboxymethylation (Figure 1a) and esterification (Figure 1b). The reactions to produce the modified starches were conducted in an automated reactor equipped with temperature and stirring control, following standardized procedures previously reported: carboxymethylation by Wang et al.^19^ and esterification by Zuo et al.^39^ For the carboxymethylation process, 8.0 g of soluble starch and 4.0 g of sodium hydroxide were added to 100 mL of ethanol and stirred at 50 °C for 1 h to dissolve. Subsequently, 4.33 g of monochloroacetic acid was added to start the carboxymethylation reaction, which was allowed to proceed for 4 h. Upon completion, the product was vacuum filtered, dried at 60 °C for 48 h, and stored at room temperature in a desiccator.^19^ The esterification reaction was conducted using a dry method in the same automated reactor. Soluble starch was mixed with maleic anhydride in a 2:1 mass ratio and the solids were mechanically stirred at 80 °C for 3.5 h. The resulting product was washed with acetone to remove unreacted maleic anhydride, filtered, dried at 60 °C for 48 h, and stored at room temperature in a desiccator.^39^

**Figure 1:**
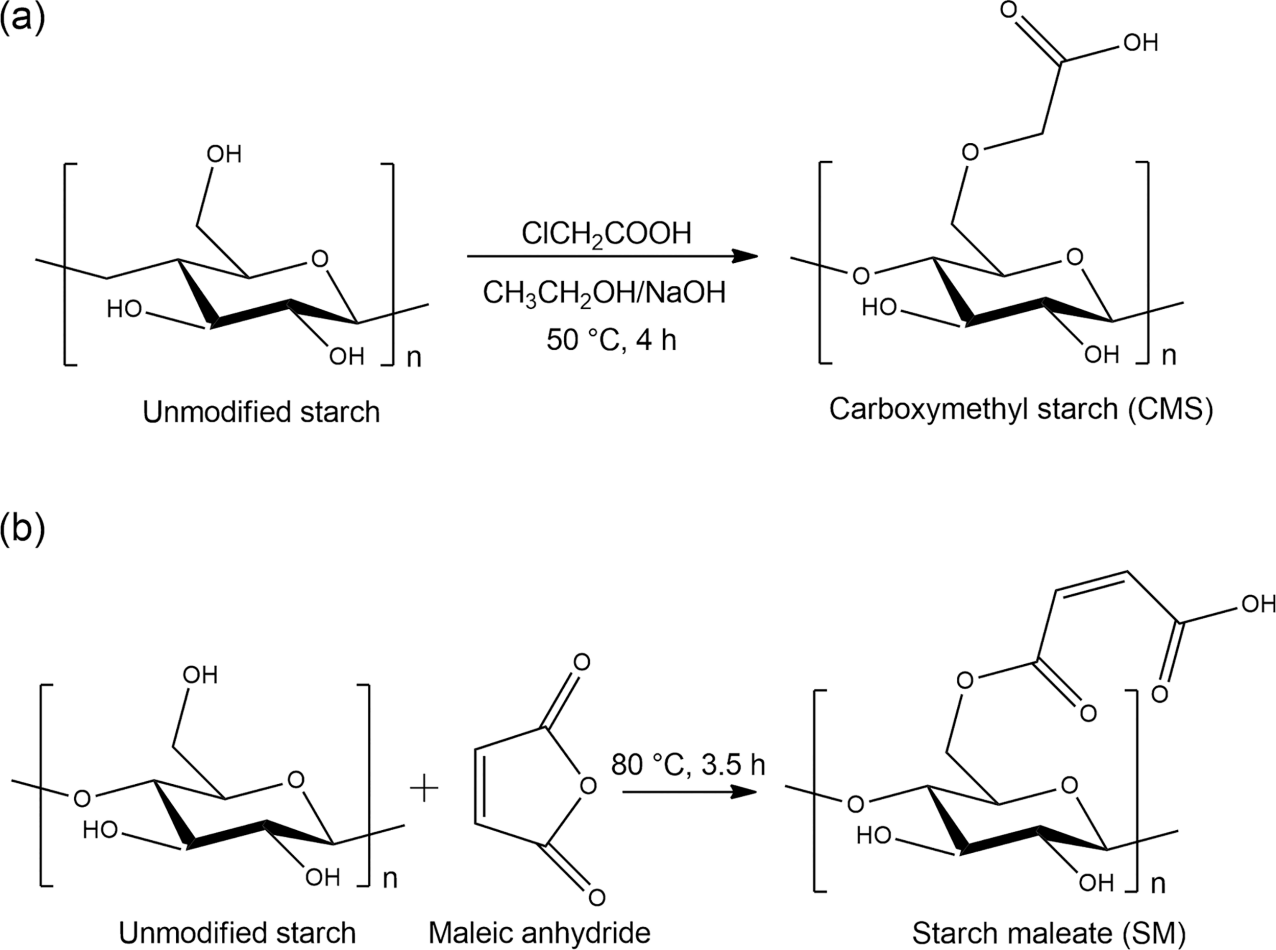
Starch modification reactions. (a) Carboxymethylation and (b) esterification.

### Characterization of the Modified Starches and Determination of the Degree of Substitution

The unmodified and modified starches were characterized by Fourier-transform infrared spectroscopy with attenuated total reflectance (FTIR-ATR). The FTIR-ATR analysis was conducted using a Bruker Tensor 27 FTIR spectrometer (Billerica, USA) at room temperature from 4000 to 400 cm^–1^ with a resolution of 4 cm^–1^. To determine the degree of substitution (DS), 1.0 g of the product was added to a mixture of 10 mL of ethanol 75% vol and 10 mL of sodium hydroxide 0.5 mol L^–1^, which was warmed at 30 °C to dissolve. The excess of sodium hydroxide was then back-titrated with a 0.5 mol L^–1^ standard hydrochloric acid using phenolphthalein as an indicator. The same procedure was conducted for the unmodified starch and the DS was calculated using eqs 1 and 2.^39^

$$W_{S}=\frac{M_{S}C_{\mathrm{HCl}}(V_{0}-V_{1})}{1000(2W)}\times 100$$ 1

$$\mathrm{DS}=\frac{MW_{S}}{M_{S}(100-W_{S})}$$ 2

Where *W*_S_ is the substituent content (carboxymethyl group or maleic anhydride) in %w/w, *W* is the mass of the modified starch in g, *M*_S_ is the molar mass of the substituent (58 g mol^–1^ for the carboxymethyl group and 98 g mol^–1^ for maleic anhydride), *M* is the molar mass of the anhydrous glucose unit (162 g mol^–1^), *C*_HCl_ is the molar concentration of the hydrochloric acid (0.5 mol L^–1^), *V*_0_ is the volume of hydrochloric acid consumed in the titration of the unmodified starch in mL, and *V*_1_ is the volume of hydrochloric acid consumed in the titration of the modified starch in mL. The degree of substitution of both modified starches was determined by triplicate.

### Tube-Blocking Tests

The performance of the modified starches in inhibiting calcium carbonate scale formation was evaluated using a tube-blocking test protocol conducted in a dynamic scale loop (DSL) system. The DSL system operated by pumping two aqueous solutions of calcium chloride and sodium bicarbonate through a test coil. Scale formation was monitored by detecting a pressure increase of more than 0.5 psi between the inlet and outlet of the test coil, measured by a pressure transducer. The effectiveness of the product was indicated by the absence of a 0.5 psi pressure increase for a duration equivalent to three times the scaling time observed in the uninhibited test, or for a total of 1 h, whichever was longer. The product concentration that meets this criterion is referred to as the minimum inhibitory concentration (MIC). The MIC was determined in duplicate to confirm the reproducibility of the results. The concentrations of the aqueous solutions before mixing was 1080 mg L^–1^ of calcium ions and 2178 mg L^–1^ of bicarbonate ions, with 17,500 mg L^–1^ of sodium chloride in both solutions. Various concentrations of the modified starches were tested by adding the desired amount to the sodium bicarbonate solution to determine the MIC. The experimental conditions for the tube-blocking tests were a flow rate of 5.000 mL min^–1^ for each solution, pH 7.0, 80 °C, and 10 bar. This procedure was also used to evaluate the unmodified starch and a commercial polymeric scale inhibitor. All tube-blocking tests were conducted in duplicate.

### Batch Precipitation Experiments and Characterization of the Formed Solids

Precipitation batch experiments were conducted in an automated reactor at 80 °C under magnetic stirring at 200 rpm. Sodium bicarbonate solution containing the modified starch at its determined MIC was mixed with calcium chloride solution. The composition of these solutions was identical to those used in the tube-blocking tests. After mixing, the system was maintained under the same experimental conditions for 1 h to allow precipitation. The resulting solids were vacuum filtered, washed with ethanol, and dried at 50 °C for 1 h. The solids formed in the absence or presence of the modified starches were characterized using Scanning Electron Microscopy (SEM) and X-ray diffraction (XRD) to investigate possible inhibition mechanisms.

The SEM analysis was conducted using a Phenom-Pro scanning electron microscope (Waltham, USA) to determine the surface morphology. Each sample of the powdered solids was placed on a conducting carbon pad (Plano GmbH) and subjected to beam scan mapping at an acceleration voltage of 15 kV. The XRD analysis was conducted using a Bruker-AXS D8 Advance Eco diffractometer to determine the crystalline phases of the samples. The measurements were performed using Cu Kα radiation (λ = 1.5406 Å) generated at 40 kV and 25 mA. Diffraction patterns were recorded over a Bragg angle (2θ) range of 5–80°, with a step size of 0.01° in continuous mode and a counting time of 92 s per step. The instrument was equipped with a state-of-the-art LynxEye XE position-sensitive linear detector based on silicon drift technology with energy discrimination. Qualitative phase identification was carried out by matching the obtained diffraction patterns with standard reference data from the Joint Committee on Powder Diffraction Standards (JCPDS) database using appropriate analysis software. The degree of crystallinity of the samples was evaluated from the XRD patterns by analyzing the characteristic reflections of the main calcium carbonate polymorphs. The (100) reflection of vaterite at 24.9°, the (111) reflection of aragonite at 26.2°, and the (104) reflection of calcite at 29.4° were selected as representative peaks for each polymorph. For each of these reflections, the crystalline contribution (*I*_net_) was calculated by subtracting the local amorphous background intensity at the adjacent valley (*I*_am_) from the measured peak maximum (*I*_peak_), following eq 3.

$$I_{\mathrm{net}}(hkl)=I_{\mathrm{peak}}(hkl)-I_{\mathrm{am}}(hkl)$$ 3

An overall index of crystallinity of each sample was then estimated as the ratio between the sum of all net crystalline intensities and the sum of the corresponding peak intensities (eq 4).

$$\mathrm{CI}=\frac{\sum I_{\mathrm{net}}(hkl)}{\sum I_{\mathrm{peak}}(hkl)}$$ 4

Finally, the relative predominance of the different polymorphs was assessed by comparing the net intensities of their characteristic reflections through intensity ratios, namely calcite to vaterite, calcite to aragonite, and vaterite to aragonite (eqs 5–7). These ratios provide a comparative measure of the distribution of crystalline forms within the samples without implying absolute phase quantification.

$$R_{C/V}=\frac{I_{\mathrm{net}}^{\mathrm{Calcite}(104)}}{I_{\mathrm{net}}^{\mathrm{Vaterite}(100)}}$$ 5

$$R_{C/A}=\frac{I_{\mathrm{net}}^{\mathrm{Calcite}(104)}}{I_{\mathrm{net}}^{\mathrm{Aragonite}(111)}}$$ 6

$$R_{V/A}=\frac{I_{\mathrm{net}}^{\mathrm{Vaterite}(100)}}{I_{\mathrm{net}}^{\mathrm{Aragonite}(111)}}$$ 7

## Results and Discussion

Figure 2 presents the FTIR-ATR spectra of the unmodified and modified starches. For the SM, two distinct absorption bands appear at 1710 and 1640 cm^–1^. These bands correspond to the stretching vibration of the C=O bond in the ester and carboxylic acid groups, respectively. For the CMS, in turn, only a band at 1600 cm^–1^ was observed, related to the asymmetric stretch of the carboxylate group.^40,41^

**Figure 2:**
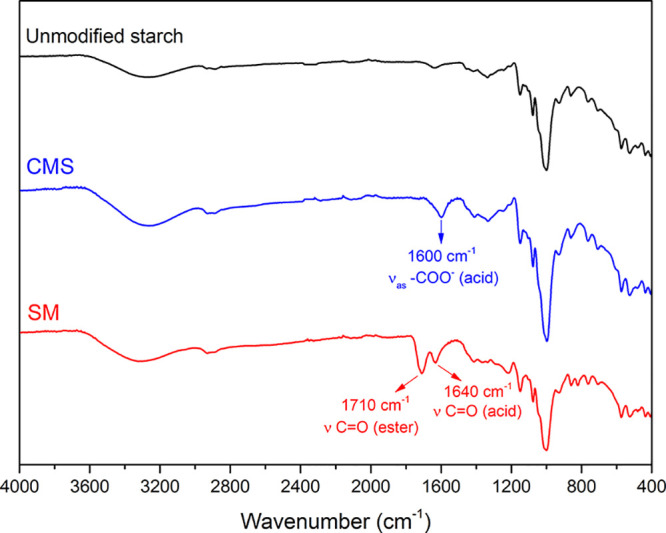
FTIR-ATR spectra of unmodified and modified starches.

To quantitatively evaluate the extent of the chemical modification, the DSs were determined via titration. The results, calculated using eqs 1 and 2, are shown in Figure 3. The DS is a key parameter in the chemical modification of starch. It refers to the average number of hydroxyl groups on each glucose unit in the starch molecule that are replaced. The maximum DS is 3, as each glucose unit has three hydroxyl groups available for substitution. Higher DS values are typically associated with improvements in the properties of the modified starch for its intended application.^42^

**Figure 3:**
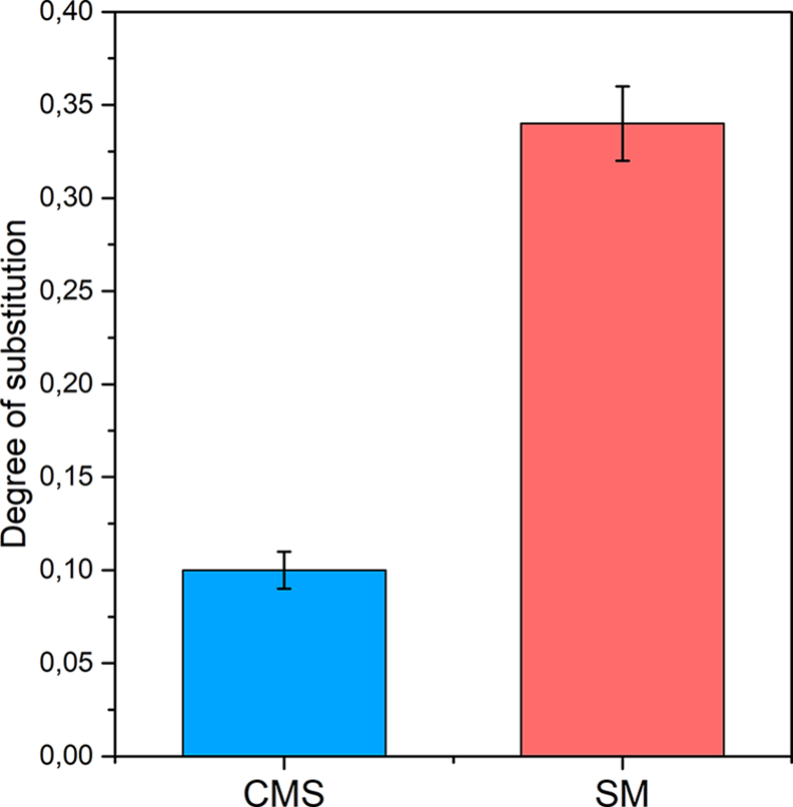
Degree of substitution (DS) of the modified starches.

The DS of the SM was higher than that of the CMS. The DS of the CMS was 0.10 ± 0.01, consistent with the value reported by Wang et al. for CMS synthesized using a 0.1:1.0 molar ratio of chloroacetic acid to starch. In their study, this product achieved a scale inhibition efficiency of 13.03% at a concentration of 60 mg L^–1^ under static test conditions (70 °C, pH 8.0, with 200 mg L^–1^of Ca^2+^ and 305 mg L^–1^ of HCO_3_^–^). Moreover, they observed that the inhibition efficiency increased with a higher DS, reaching 89.80% at a DS of 0.95. This trend highlights the role of the carboxymethyl substitution: increased substitution introduces more carboxyl groups, which enhances anionic charge density and promotes stronger interactions with calcium ions.^19^ Therefore, the higher DS of SM (0.34 ± 0.02) suggests that it may offer improved scale inhibition performance compared to CMS.

A similar relationship between DS and inhibition efficiency in static tests was previously reported by Boels and Witkamp (2011) for carboxymethyl inulin. In that study, carboxymethyl inulin with 2.5 carboxylate groups per fructose unit outperformed the variant with 2.0 carboxylate groups, highlighting the important role of negative charge density in effective scale inhibition under the studied conditions (25 °C, pH 8.1–8.4, 294–588 mg L^–1^ of CaCl_2_, 336 mg L^–1^ of NaHCO_3_^–^, and 6560.5 mg L^–1^ of KCl).^43^ A higher DS is also desirable to enhance the water solubility of biopolymers. Macedo et al. synthesized carboxymethyl chitosan with DS ranging from 0.40 to 0.60 to obtain a water-soluble polymer across a broad pH range, containing a substantial number of polar and chelating groups. In dynamic tests conducted at 70 °C, 69 bar, and a flow rate of 10 mL min^–1^, they reported a minimum inhibitory concentration (MIC) of 170 mg L^–1^ under conditions simulating oilfield brines (2231 mg L^–1^ of Na^+^, 85 mg L^–1^ of K^+^, 152 mg L^–1^ of Ca^2+^, 33 mg L^–1^ of Mg^2+^, 1000 of NaHCO_3_^–^, and 2686 of Cl^–^).

The effect of a higher DS on enhancing scale inhibition was corroborated by the tube-blocking test results presented in Figure 4. The MIC of the SM was 125 mg L^–1^, which is lower than the 150 mg L^–1^ observed for CMS. In addition to the higher DS, as the substituent is different, other steric effects may presumably contribute to the enhanced performance of SM. As illustrated in Figure 1, the SM contains carboxylic and ester groups, whereas CMS has carboxylic and ether groups. The ester functionality in SM provides an additional site to interact with scaling ions, nuclei, and solids, thereby contributing to its superior scale inhibition.^44,45^ Although weaker than interactions involving carboxylates, carbonyl-calcium coordination is generally stronger than ether-calcium interactions. Computational studies support this trend: Chen et al. reported higher binding energies on the (110) and (104) faces of calcium carbonate for oxidized starch compared to carboxymethyl cellulose, with even stronger interactions for poly­(aspartic acid).^46^ Similarly, Zuo et al. reported that the binding energies of poly­(acrylic acid) on the (104) and (110) surfaces of calcite were 106.2 and 141.3 kcal mol^–1^, respectively, values that were significantly higher than the corresponding 69.7 and 86.2 kcal mol^–1^ obtained for poly­(epoxysuccinic acid). Considering that poly­(acrylic acid) contains more free carboxylic/carbonyl groups, whereas poly­(epoxysuccinic acid) has fewer or more constrained carbonyls, these findings support the observation that polymers with more carbonyl groups interact more strongly with calcium carbonate surfaces and exhibit superior scale inhibition.^47^

**Figure 4:**
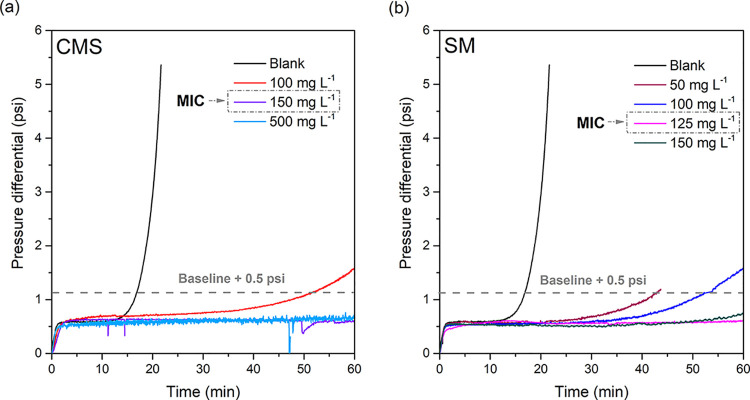
Tube-blocking tests results. (a) CMS and (b) SM.

Although the scale inhibition performances of CMS and SM are relatively similar, it is important to emphasize that both modified starches significantly outperformed the unmodified starch. As shown in Figure 5, the MIC of the unmodified starch was 500 mg L^–1^, whereas the MICs for CMS and SM were 150 and 125 mg L^–1^, respectively. These values represent reductions of 70 and 75%, respectively, highlighting the substantial improvement in inhibition efficiency achieved through chemical modification. This reduction in the MIC may positively impact the future development of commercial scale inhibitors based on these modified biodegradable polymers by lowering required dosages, potentially reducing formulation costs, minimizing possible compatibility issues with other components, and extending shelf life.

**Figure 5:**
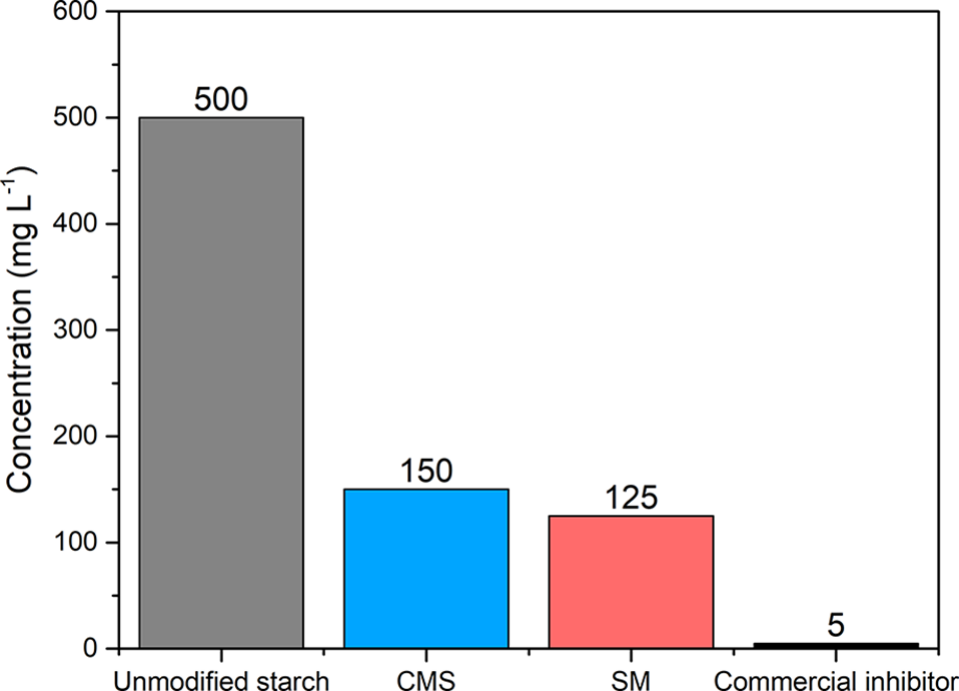
Comparison of the MICs of unmodified and modified starches.

Figure 5 also shows that the MICs of modified starches are higher than that of a commercial polymeric inhibitor evaluated under same experimental conditions. However, caution is required when directly comparing the molecules investigated with a commercial inhibitor. Commercial inhibitors often undergo further stages of formulation, in which the active compound can be combined with other additives that may enhance the overall inhibitory efficiency. Therefore, a more appropriate benchmark would be the MIC, determined by tube-blocking tests, for other noncommercial green scale inhibitors, in particular, modified biodegradable polymers applied to petroleum production.^22,48^ Some studies on green scale inhibitors for petroleum production, however, do not report the MIC but rather the fail inhibitor concentration (FIC), defined as the concentration at which scale starts to form when the inhibitor concentration is reduced. A lower FIC indicates that less inhibitor is required to prevent scale and thus reflects better performance. Consequently, MIC values are generally higher than FIC values.^10,29^ As mentioned in the introduction, most studies on modified biodegradable polymers have focused on water treatment and are typically conducted under static conditions at atmospheric pressure. Table 2 summarizes selected studies reporting the MIC and FIC values of modified biodegradable polymers investigated as scale inhibitors in conditions relevant for petroleum production.

**Table 2:** Reported MIC or FIC Values of Modified Biodegradable Polymers for Scale Inhibition in Petroleum Production

inhibitor	MIC or FIC (mg L^–1^)	temperature (°C)	pH	[Ca^2+^] (mg L^–1^)	[HCO_3_^–^] (mg L^–1^)	reference
carboxymethyl starch	150^a^	80	7.0	540	1089	this study
starch maleate	125^a^	80	7.0	540	1089	this study
carboxymethyl chitosan	170^a^	70	8.2	152	1000	Macedo et al.^18^
carboxymethyl cellulose + hydroxyethyl cellulose	10 + 200^a^	100	7.0	792	1330	Fernandes et al.^27^
carboxymethyl inuline	5^b^	100		724	500	Mady et al.^29^
phosphonated chitosan	10^b^	100		724	500	Mady et al.^29^
oxidized lignosulfonate	10–5^b^			1020	500	Ganguly et al.^10^

a MIC.

b FIC.

Observe that the experimental conditions presented in Table 2 differ for each inhibitor, including brine composition, pH, and temperature, which makes direct comparison challenging. Nevertheless, the MIC values reported in this study fall within a range similar those of carboxymethyl chitosan and hydroxyethyl cellulose. Moreover, the relatively low degree of substitution of CMS and SM (0.10/3.00 and 0.34/3.00, respectively) contributes to their advantages in terms of cost and biodegradability, as their main component is soluble starch. Starch is an abundant, inexpensive, and biodegradable polymer that can even be recovered from byproducts and effluents.^49,50^ Indeed, the soluble starch used in this study costs approximately 9 US dollars per kilogram. Importantly, the high biodegradability of starch is preserved even in modified forms, including copolymers and blends, making it a promising candidate for the development of green scale inhibitors. Wu (2003) evaluated the properties of polycaprolactone-starch and maleic anhydride-grafted-polycaprolactone-starch blends, including their biodegradability. The weight loss of both blends when buried in soil confirmed biodegradation, even at high levels of starch substitution.^51^ Nagasawa et al. synthesized a cross-linked CMS hydrogel by irradiation and reported ∼40% biodegradation under controlled composting after 2 weeks, a rate faster than that of standard cellulose powder.^52^ Zuo et al. produced a composite SM and polylactic acid and investigated its natural aging degradation using the soil burial method. The degradation rate of the SM/polylactic acid composite was comparable to that of native starch/polylactic acid, with the SM/polylactic acid composite showing even slightly higher mass loss after 30 days. This effect was attributed to the destruction of the crystalline structure of starch during esterification, which facilitated water and microorganism penetration into starch molecule.^53^

Precipitation batch experiments were conducted to better understand the enhanced performance of the modified starches. The calcium carbonate solids were characterized using SEM and XRD. Figure 6 presents the SEM images, comparing the morphologies of the solids formed in the absence of inhibitors (blank) and in the presence of the modified starches. Figure 6a shows that the solids formed in the blank experiment primarily consist of aragonite, which appears as rod- or needle-like structures. In addition, smaller prismatic particles of calcite and a few larger dendritic flower-like structures of vaterite are also observed. At the experimental temperature of 80 °C, aragonite is the most stable crystalline phase of calcium carbonate.^54^ Vaterite, on the other hand, is a metastable phase, and its characteristic dendritic flower-like morphology may result from the relatively low stirring rate used during the experiment (200 rpm). The presence of calcite may be attributed to the partial transformation of vaterite into this more stable phase, following the Ostwald phase transition, as vaterite formation is favored at lower temperatures (around 40–60 °C).^55,56^

**Figure 6:**
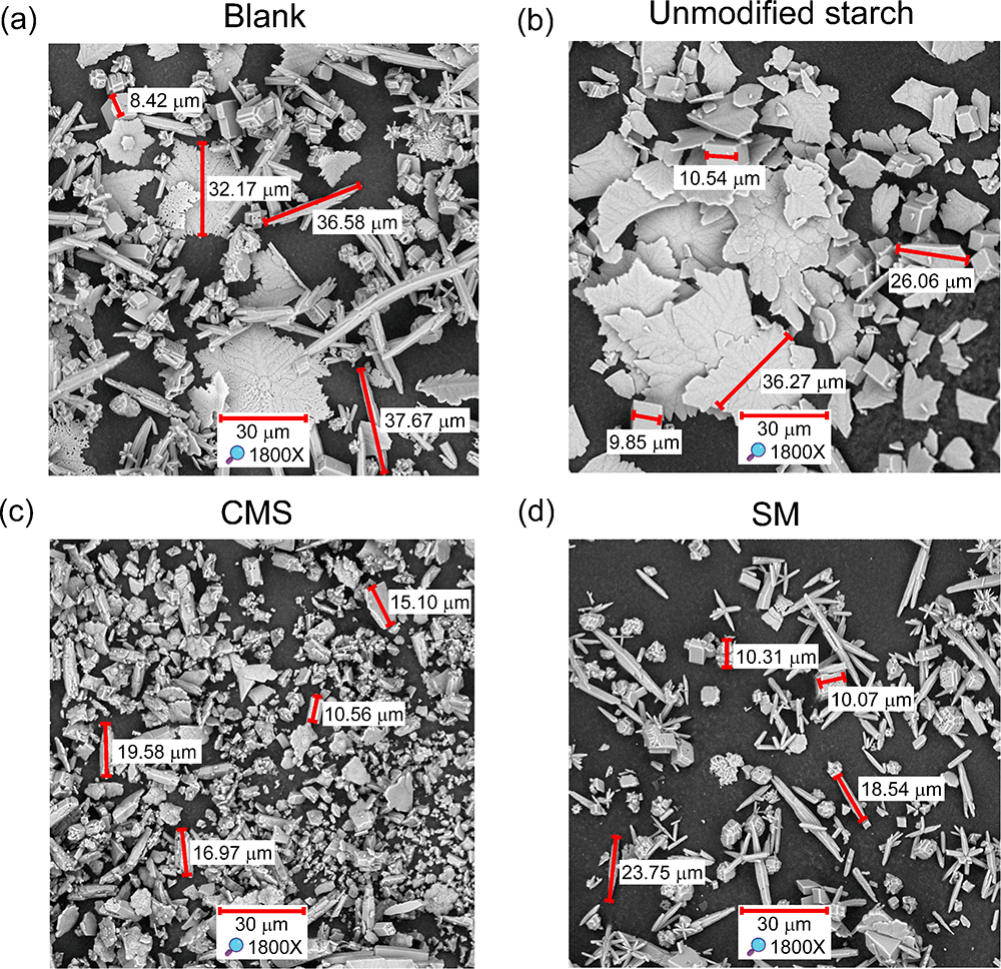
SEM images of calcium carbonate solids formed in the presence of unmodified and modified starches. (a) Blank, (b) unmodified starch, (c) CMS, and (d) SM.

Figure 6b shows that the solids formed in the presence of unmodified starch consist of distorted cubic particles of calcite and dendritic flower-like structures of vaterite, with particle sizes comparable to those observed in the blank experiment. This observation is consistent with a previous study, in which was reported the stabilization of calcite in the presence of various carbohydrates and the formation of both calcite and vaterite in the presence of soluble starch.^23^ The formation of large, well-defined particles in the presence of unmodified starch may explain its higher MIC, as these structures suggest limited interaction with the particles during the crystal growth process.

Figure 6c shows that the CMS promotes the formation of irregular and smaller particles. Many of these particles appear flattened, with irregular edges and poorly defined facets, suggesting inhibited crystal growth along specific planes. Elongated aragonite-type structures are notably scarce, suggesting that CMS suppresses aragonite crystalline habit growth. Although some calcite particles are present, they appear rounded or fractured, likely due to CMS interfering with the development of typical crystal faces. Additionally, numerous small grain clusters are observed, which may correspond to an amorphous phase. Wang et al. similarly reported that CMS disrupted the normal growth process of calcium carbonate crystals and proposed that this effect may result from the irreversible adsorption of CMS at active growth sites on the crystal surface.^19^

Figure 6d reveals a distinct crystal growth behavior in the presence of SM compared to CMS, indicating a different mode of interaction between SM and calcium carbonate. The image displays prominent rod- and needle-shaped particles, along with starburst clusters, suggesting that SM more effectively stabilizes the aragonite phase than CMS. Additionally, well-defined prismatic calcite particles are present, while amorphous clusters and vaterite structures appear less frequently than in the CMS sample. Notably, SM appears to induce a reduction in the particle size of the precipitates when compared to the blank experiment. This apparent reduction in particle size may explain its lower MIC.

To corroborate the morphological observations from the SEM images, the calcium carbonate solids were also analyzed using XRD. Figure 7 presents the XRD diffractograms, with marked peaks for each of the polymorphs.

**Figure 7:**
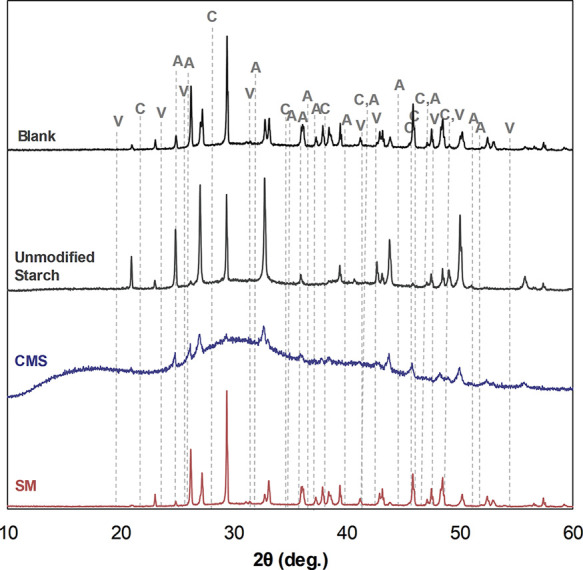
XRD diffractograms of calcium carbonate precipitated in the presence of the unmodified and modified starches.

The characteristic peaks in the XRD confirm the presence of aragonite, calcite, and vaterite in the absence of inhibitors. The unmodified starch promotes the formation of calcite and vaterite only, which may show that this molecule stabilizes these phases in contrast to aragonite. SM, on the other hand, although influencing crystal size, does not influence the polymorphs phase stabilization, as all three calcium carbonate forms are seen. As for the CMS, the XRD patterns exhibit broad diffuses halos around 10–30°, lacking the sharp and well-defined peaks observed in the other samples. This feature is characteristic of an amorphous material, supporting the findings in the SEM images. Therefore, the CMS not only affects crystal growth, but also affects the stabilization of calcium carbonate polymorphs. Wang et al. also reported that CMS disturbs the normal growth process of calcium carbonate crystals. They attributed the mechanism for controlling scale growth to the irreversible adsorption of CMS onto active growth sites of crystals. Moreover, the calcium carbonate became more irregular, coarse, and amorphous as the CMS substitution degree increased.^19^

Overall, the SEM and XDR results are interesting and show that even though the CMS and SM represent modification in the starch molecule, leaving it with a carboxylic acidic group, and their inhibition efficiency are similar in the tube blocking test, their behavior and mechanism of action during calcium carbonate are different. CMS inhibits crystal growth by distorting particle shape and size, while SM primarily reduces particle size with less morphological distortion. This qualitative interpretation is supported by a semiquantitative evaluation of the XRD data, as shown in Table 3.

**Table 3:** Index of Crystallinity and Intensity Ratios Derived from XRD Patterns of Calcium Carbonate Solids

sample	*I*_net_^Vaterite^	*I*_net_^Aragonite^	*I*_net_^calcite^	CI	*R*_C/V_	*R*_C/A_	*R*_V/A_
blank	994	3804	6480	0.97	6.52	1.70	0.26
unmodified starch	2962	659	5239	0.95	1.77	7.95	4.50
CMS	728	1378	1196	0.47	1.64	0.87	0.53
SM	563	4939	9841	0.99	17.48	1.99	0.11

The index of crystallinity was higher than 0.95 for the blank, unmodified starch, and SM, confirming that these samples are predominantly crystalline, while CMS exhibited a much lower value (0.47), consistent with its broad amorphous halo. The relative intensity ratios between the selected reflections further highlight these differences: Blank and SM are dominated by calcite, unmodified starch favors calcite and vaterite at the expense of aragonite, and CMS shows overall ratios close to unity, indicating no predominant crystalline polymorph. These results suggest that CMS does not merely inhibit crystal growth but fundamentally suppresses long-range order and the stabilization of specific polymorphs, whereas unmodified starch and SM selectively stabilize calcite/vaterite and aragonite, respectively, in agreement with the morphological signatures observed in the SEM analysis.

## Conclusions

This work demonstrates that chemically modified soluble starches, specifically carboxymethyl starch (CMS) and starch maleate (SM), exhibit significant potential as green scale inhibitors for calcium carbonate under conditions relevant to petroleum production. Both modified biodegradable polymers considerably outperformed the unmodified starch, with SM achieving the lowest minimum inhibitory concentration of 125 mg L^–1^. The superior performance of SM compared to CMS can be attributed to its higher degree of substitution. Scanning electron microscopy analysis indicated that the modified starches disrupted typical crystal growth patterns, leading to distorted morphologies and reduced particle sizes, which may explain their improved scale inhibition performance compared to the unmodified starch. Furthermore, the solvent-free esterification route with maleic anhydride offers an operationally simplified and potentially scalable modification pathway. These findings support the feasibility of starch-based polymers as cost-effective and environmentally friendly alternatives to conventional scale inhibitors, aligning with current industry efforts to minimize environmental impact while ensuring flow assurance in oilfield operations. Future work should include a biodegradability test, evaluation of compatibility with other oilfield chemicals, formulation of a commercial scale inhibitor, and field trails. Such investigations will provide a clearer understanding of the industrial applicability of CMS and SM, paving the way for their adoption as sustainable scale inhibition solutions in petroleum production.

## ABBREVIATIONS

CMS: carboxymethyl starch
DS: degree of substitution
DSL: dynamic scale loop
FIC: fail inhibitory concentration
FTIR-ATR: Fourier-transform infrared spectroscopy with attenuated total reflectance
JCPDS: Joint Committee on Powder Diffraction Standards
MIC: minimum inhibitory concentration
SEM: scanning electron microscopy
SM: starch maleate
XRD: X-ray diffraction
